# Prolonged culturing of iPSC-derived brain endothelial-like cells is associated with quiescence, downregulation of glycolysis, and resistance to disruption by an Alzheimer’s brain milieu

**DOI:** 10.1186/s12987-022-00307-1

**Published:** 2022-02-05

**Authors:** Lindsey M. Williams, Takashi Fujimoto, Riley R. Weaver, Aric F. Logsdon, Kira M. Evitts, Jessica E. Young, William A. Banks, Michelle A. Erickson

**Affiliations:** 1 Geriatrics Research Education and Clinical Center (GRECC), Veterans Affairs Puget Sound Health Care System, Seattle, WA USA; 2 Department of Neurosurgery, Nagasaki University Graduate School of Biomedical Sciences, Nagasaki, Nagasaki Japan; 3 Department of Medicine, Division of Gerontology and Geriatric Medicine, University of Washington School of Medicine, Seattle, WA USA; 4 Department of Bioengineering, University of Washington, Seattle, WA USA; 5 Institute for Stem Cell and Regenerative Medicine, University of Washington, Seattle, WA USA; 6 Department of Laboratory Medicine and Pathology, University of Washington, Seattle, WA USA

**Keywords:** Blood–brain barrier (BBB), Human induced pluripotent stem cells (iPSCs), Quiescence, Glycolysis, Glucose transporter-1 (GLUT1), Tight junction protein (TJP), Alzheimer’s disease (AD)

## Abstract

**Background:**

Human induced pluripotent stem cell (hiPSC)-derived brain endothelial-like cells (iBECs) are a robust, scalable, and translatable model of the human blood–brain barrier (BBB). Prior works have shown that high transendothelial electrical resistance (TEER) persists in iBECs for at least 2 weeks, emphasizing the utility of the model for longer term studies. However, most studies evaluate iBECs within the first few days of subculture, and little is known about their proliferative state, which could influence their functions. In this study, we characterized iBEC proliferative state in relation to key BBB properties at early (2 days) and late (9 days) post-subculture time points.

**Methods:**

hiPSCs were differentiated into iBECs using fully defined, serum-free medium. The proportion of proliferating cells was determined by BrdU assays. We evaluated TEER, expression of glycolysis enzymes and tight and adherens junction proteins (TJP and AJP), and glucose transporter-1 (GLUT1) function by immunoblotting, immunofluorescence, and quantifying radiolabeled tracer permeabilities. We also compared barrier disruption in response to TNF-α and conditioned medium (CM) from hiPSC-derived neurons harboring the Alzheimer’s disease (AD)-causing Swedish mutation (APP^Swe/+^).

**Results:**

A significant decline in iBEC proliferation over time in culture was accompanied by adoption of a more quiescent endothelial metabolic state, indicated by downregulation of glycolysis-related proteins and upregulation GLUT1. Interestingly, upregulation of GLUT1 was associated with reduced glucose transport rates in more quiescent iBECs. We also found significant decreases in claudin-5 (CLDN5) and vascular endothelial-cadherin (VE-Cad) and a trend toward a decrease in platelet endothelial cell adhesion molecule-1 (PECAM-1), whereas zona occludens-1 (ZO-1) increased and occludin (OCLN) remained unchanged. Despite differences in TJP and AJP expression, there was no difference in mean TEER on day 2 vs. day 9. TNF-α induced disruption irrespective of iBEC proliferative state. Conversely, APP^Swe/+^ CM disrupted only proliferating iBEC monolayers.

**Conclusion:**

iBECs can be used to study responses to disease-relevant stimuli in proliferating vs. more quiescent endothelial cell states, which may provide insight into BBB vulnerabilities in contexts of development, brain injury, and neurodegenerative disease.

**Supplementary Information:**

The online version contains supplementary material available at 10.1186/s12987-022-00307-1.

## Introduction

The vascular blood–brain barrier (BBB) is a vital interface that limits the unregulated transfer of circulating substances into the brain and facilitates the regulated transport of substances such as ions, nutrients, and signaling molecules that are essential for CNS homeostasis [1]. The BBB is primarily comprised of highly specialized brain endothelial cells (BECs) that confer both barrier and interface functions. The barrier functions are attributed, in part, to tight and adherens junction proteins (TJP and AJP), which effectively seal the space between neighboring BECs to prevent paracellular diffusion [2]. Suppression of transcellular leakage via pinocytic vesicles, expression of efflux transporters, and enzymatic degradation of blood-derived substances also contribute to the barrier properties of BECs [3, 4]. The interface functions of the BBB include transporter-mediated passage of nutritive and regulatory substances, secretory functions, and the ability of BECs to modulate their activities in response to signals from the blood or brain compartments. Each of these functions can facilitate communication between the brain and the periphery, thus regulating CNS activities [5].

There is mounting interest in understanding the physiology of the BBB and its pathological changes in disease contexts. The development of in vitro models of the human BBB that derive BEC-like cells (iBECs) from human induced pluripotent stem cells (hiPSCs) offers a robust platform for studying many aspects of BBB function, including interactions between BECs and other cell types of the neurovascular unit (pericytes, astrocytes, neurons) [6], genetic contributions to BBB dysfunction, and mechanisms of BBB dysfunction in neurological diseases [7–11]. iBECs express TJPs and AJPs, and functional nutrient and efflux transport systems [12–15]. One major benefit of iBECs is that they develop high trans-endothelial electrical resistance (TEER) and low permeability to inert tracers, approximating in vivo barrier properties. This allows for the study of BBB transport systems with minimal confounds of leakage. In most studies, iBECs have been characterized and tested two days after subculturing the differentiated cells (post-subculture), though it has been shown that they retain strong barrier properties for at least two weeks [14, 15]. The preservation of high TEER in iBECs suggests that they can be used for longer in vitro studies. However, further characterization of the expressional and functional properties of iBECs at extended post-subculture timepoints is needed.

In this study, we characterized the phenotypic changes that occur over time in iBECs derived from the GM25256 hiPSC line by comparing protein expression and functional outcome measures at day 2 and day 9 post-subculture. These time points were selected to reflect a time conventionally used in the literature, day 2 post-subculture, and a later time to which GM25256-derived iBECs reliably sustained high TEER, day 9 post-subculture. We first noted that the amount of cellular proliferation was significantly lower in iBECs subcultured for nine days vs. two days. Subsequently, we characterized how key aspects of the BBB phenotype are altered in iBECs’ more quiescent state. We focused on: (1) metabolic markers of endothelial quiescence, because an altered relation between glucose metabolism and transendothelial glucose transport may be important to consider when using iBECs to study dysfunctional GLUT1-mediated glucose transport at the BBB in Alzheimer’s disease (AD) [16–18], (2) markers of BBB integrity, such as TEER and expression of TJP and AJP, because BEC proliferation has been associated with BBB leakiness [19, 20], and (3) responses to inflammatory and AD-associated insults, which are insults that can happen concurrently with increased BEC proliferation [21, 22]. Our findings indicate that length of subculture influences the baseline phenotypes of iBECs, as well as their responses to conditioned medium (CM) from hiPSC-neurons harboring the familial AD-causing Swedish mutation. These findings provide new insight into the relations between BEC proliferative state and BBB functions at baseline and in response to disease-related insults.

## Methods

### Derivation of brain endothelial-like cells (iBECs) from hiPSCs and TEER measurements

iBECs were derived using the method of Neal et al. [15]. Human iPSCs (hiPSCs) from the GM25256 line (Coriell Institute) were maintained on plates coated with Matrigel (Corning, cat no. 356230) in E8 Flex medium (Thermo Fisher Scientific, cat no. A28585-01). The day before differentiation was initiated, hiPSCs were dissociated into single cells with Accutase (Thermo Fisher Scientific, cat no. A1110501) and plated onto Matrigel-coated plates at a density of 15 × 10^3^ cells/well in E8 Flex medium supplemented with 10 µM Rho-associated protein kinase (ROCK) inhibitor Y-27632 (R&D Systems, cat no. 1254). To initiate differentiation, the medium was changed to E6 (Thermo Fisher Scientific, cat no. A1516401) and E6 medium changes continued daily for 3 more days. Then, the medium was changed to human endothelial serum-free medium (HESFM, Thermo Fisher Scientific, cat no. 11111044) supplemented with 20 ng/mL basic fibroblast growth factor (bFGF; Peprotech, cat no. 100-18B), 10 µM retinoic acid (RA; Sigma, cat no. R2625), and 1% B27 supplement (Thermo Fisher Scientific, cat no. 17504044). After two days, iBECs were dissociated with Accutase and subcultured onto 24-well transwell permeable inserts (Corning, cat no. 3470) or tissue culture plates (Corning, cat no. 3513, 3548) coated with 1 mg/mL Collagen IV (Sigma, cat no. C5533) and 5 mM Fibronectin (Sigma, cat no. F1141) in HESFM + 20 ng/mL bFGF, 10 µM RA, and 1% B27 (day 0). 24 h later, the medium was changed to HESFM + 1% B27 without bFGF or RA, and resistance ($\Omega$) values for monolayers of iBECs seeded on transwells were obtained for day 1 using an EVOM2 Voltohmmeter (World Precision Instruments, Sarasota Florida) coupled to an ENDOHM cup chamber. Transendothelial electrical resistance (TEER) was calculated by subtracting the resistance ($\Omega$) value of a blank transwell and multiplying by the transwell surface area (0.33 cm^2^). TEER measurements occurred daily to confirm the integrity of the monolayers. Experiments were only conducted on iBECs with TEER > 1000 $\Omega$*cm^2^.

### Immunofluorescence analyses

iBECs plated on 48-well plates were washed once with PBS (Thermo Fisher Scientific, cat no. 70011044) and fixed in a 1:1 methanol/acetone mixture for 15 min at 4 °C. Wells were washed with PBS 3 × for 5 min each, then blocked with 5% normal donkey serum (Jackson ImmumoResearch, cat no. 017-000-121) + 0.1% TX-100 (Sigma, cat no. ×100) in PBS for 1 h at RT. Wells were washed 3 × for 5 min each, then iBECs were incubated with primary antibody solutions (see Table 1 for dilutions) in phosphate-buffered normal antibody diluent (NAD) (Scytek, cat no. ABB500) overnight at 4 °C. Wells were washed 3 × for 5 min each, then incubated with secondary antibody solutions (all 1:200) and DAPI (Thermo Fisher Scientific, cat no. 62248) (1:5000) in NAD for 1 h at RT. Wells were washed 3 × for 5 min each, then imaged using a Zeiss Axiovert 7. For each differentiation, 3 wells were designated per group and 3–4 images were taken per well. Mean fluorescence intensity (MFI) values were quantified using the Zen image analysis software and were corrected for differences in cell density via normalization to DAPI MFI.

**Table 1:** Antibodies

Target antigen	Vendor	Catalog number	Dilution
5-Bromo-2'-deoxyuridine (BrdU)	abcam	ab6326	1:250
PFKFB3	Cell Signaling	13123S	1:500
GLUT1	EMD Millipore	07-1401	1:10,000
HK2	Cell Signaling	2867 T	1:1000
MCT1	Proteintech	20139-1-AP	1:10,000
CLDN5	ThermoFisher Scientific	35-2500	1:50
OCLN	ThermoFisher Scientific	33-1500	1:50
ZO-1	ThermoFisher Scientific	61-7300	1:25
PECAM-1	Sigma	P8590	1:25
VE-Cadherin	R&D Systems	AF938	1:25
$\beta$-actin $\beta$-actin, HRP-linked	Cell Signaling abcam	3700S ab49900	1:10,000 1:20,000
Anti-mouse IgG, HRP-linked	Jackson ImmunoResearch	115-035-003	1:5000
Anti-rabbit IgG, HRP-linked	Jackson ImmunoResearch	111-035-144	1:5000
AlexaFluor^®^ 488-Anti-rat IgG	ThermoFisher Scientific	A-21208	1:500
AlexaFluor^®^ 488-Anti-mouse IgG	Jackson ImmunoResearch	715-545-150	1:200
AlexaFluor^®^ 594-Anti-rabbit IgG	Jackson ImmunoResearch	711-585-152	1:200
AlexaFluor^®^ 594-Anti-goat IgG	Jackson ImmunoResearch	705-585-147	1:200

### BrdU proliferation assays

Analysis of 5-bromo-2′-deoxyuridine (BrdU) (Thermo Fisher Scientific, cat no. B23151) incorporation into the DNA was used to evaluate iBEC proliferation. For iBECs fixed on day 2, 10 µM BrdU was included with the medium change to HESFM + 1% B27 without bFGF or RA on day 1. For iBECs fixed on day 4, 5, 7, or 9, BrdU was added to a final concentration of 10 µM by diluting a 100 µM BrdU solution into the existing medium on day 3, 4, 6, or 8, respectively. 24 h after addition of 10 µM BrdU, cells were washed 1 × quickly with PBS and fixed in ice-cold 1:1 methanol/acetone fixative for 15 min. at 4 °C. Cells were permeabilized with 0.1% TX-100 in PBS for 20 min at RT. DNA strands were denatured with 2 N HCl for 10 min at RT. Then, the IFA for was performed according to the procedure outlined above.

### Protein extractions and immunoblots

iBECs plated on 12-well plates were washed in ice-cold PBS and scraped in a RIPA-like buffer (1% cytoplasmic stock, 1% NP-40, 0.5% Deoxycholate, 0.1% SDS) supplemented with protease (Sigma, cat no. P8340) and phosphatase (Sigma, cat no. P5726) inhibitors. Lysates were frozen at − 80 °C, then thawed on ice and centrifuged at 20000×*g* for 5 min at 4 °C. The supernatant was saved and protein concentrations for each cell lysate were determined by Bradford assay (Thermo Fisher Scientific, cat no. 23200) using known concentrations of bovine serum albumin to create the protein standard curve. Samples were prepared for electrophoresis by mixing with 4 × LDS Sample Buffer (Novex, cat no. NP007), 10 × Sample Reducing Agent (Novex, cat no. NP0009), and molecular biology-grade water, then denatured at 70 °C for 10 min. Protein mixtures containing 10 μg of protein were electrophoresed using ExpressPlus PAGE precast gels (GeneScript, cat no. M41210), then electrotransferred to nitrocellulose membranes (Invitrogen, cat no. IB301002) using the iBlot Dry Blotting System (Invitrogen)*.* Nonspecific binding was blocked by incubating the membrane in Tris-buffered saline 0.1% Tween-20 (TBS-T) supplemented with 5% BSA (Sigma, cat no. A7030) at RT for 45 min. Primary antibody solutions (see Table 1 for dilutions) were prepared in 5% BSA/TBS-T and membrane incubations occurred overnight at 4 °C. Membranes were washed with TBS-T three times for 5 min each, then incubated with horseradish peroxidase conjugated secondary antibodies (all 1:5000) at RT for 30 min. Then, membranes were washed with TBS-T three times for 5 min each followed by one TBS wash for 1 min. West Pico chemiluminescence reagent (Thermo Fisher Scientific, cat no. PI-34078) was applied, and bands of immunoreactive protein were visualized with the ImageQuant LAS4000 (Cytiva Life Sciences, formerly GE Healthcare Life Sciences) Western blot Imaging system or the Amersham™ ImageQuant 800 (Cytiva Life Sciences, formerly GE Healthcare Life Sciences). Quantification of the band volumes was performed with ImageQuant TL 1D Gel Analysis Software (Cytiva Life Sciences, formerly GE Healthcare Life Sciences).

### Glucose transport assays

Transwells were distributed such that the mean TEER values were approximately equal among groups. The luminal and abluminal HESFM + 1% B27 mediums were refreshed, and cells were equilibrated in the incubator for 20 min. The input medium consisted of HESFM + 1% B27 containing 0.1 µCi ^14^C-2-deoxy-d-glucose (^14^C-DG, FW 164.16 g/mol) (Perkin Elmer) and 1 million CPM ^99m^Technetium diethylenetriaminepentaacetic acid (^99m^Tc-DTPA, FW 487.21 g/mol) per transwell. As 2-deoxy-d-glucose is transported at nearly the same rate as glucose and is trapped intracellularly after hexokinase phosphorylation, it is an appropriate tracer to measure transcellular glucose transport [23]. ^99m^Tc-DTPA permeability was measured as a non-specific para- and transcellular leakage control. To evaluate the saturability of the iBEC glucose transport system, the input medium was supplemented with d(+)-Glucose (Sigma, cat no. G7528) or d-Mannitol (Sigma, cat no. M4125). BAY-876 (Tocris, cat no. 6199) was obtained as a powder and stored at − 20 °C in a glass vial. Stock solutions (2 mM) were prepared by diluting BAY-876 in DMSO (Sigma, cat no. D2650) (vehicle control) and were stored in 25 µL aliquots at − 70 °C. The input medium was supplemented BAY-876 to a final concentration of 2 µM to evaluate GLUT1 inhibition. For assays evaluating luminal-to-abluminal (blood-to-brain) transport, the 100 µL luminal (donor) chamber volume was switched to the input medium to initiate the assay, and 500 µL volumes of medium from the abluminal (receptor) chamber were collected and replaced with fresh, pre-warmed HESFM + 1% B27 medium after incubation times of 5, 10, 15, and 20 min. Of the 500 µL total volume collected, 200 µL was added to liquid scintillation vials containing 5 mL of Ecoscint™ (National Diagnostics). The vials were dark-adapted for at least 4 days. Then, the radioactivity was counted in a Tricarb beta counter (Perkin Elmer) to measure ^14^C-DG levels. The radioactivity in the remaining 300 µL was counted in a Wizard 2 gamma counter (Perkin Elmer) to measure ^99m^Tc-DTPA levels. For abluminal-to-luminal (brain-to-blood) transport assays, the 600 µL abluminal (donor) chamber volume was switched to an input medium to initiate the assay, and 90 µL volumes of medium from the luminal (receptor) chamber were collected and replaced with fresh, pre-warmed HESFM + 1% B27 medium after incubation times of 5, 10, 15, and 20 min. Of the 90 µL collection, 40 µL was counted in the beta counter and the remaining 50 µL was counted in the gamma counter. Permeability-surface area coefficient (Pe) calculations were performed according to the method of Dehouck et al. [24]. Clearance was expressed as µL of radioactive tracer transported from the donor chamber to the receptor chamber, and was calculated from the initial level of radioactivity added to the donor chamber and the final level of radioactivity in the receptor chamber:

$${\text{Clearance }}\left( {\mu {\text{L}}} \right) \, = \, \left[ {\text{C}} \right]_{{\text{C}}} \times {\text{ V}}_{{\text{C}}} /\left[ {\text{C}} \right]_{{\text{L}}} .$$

where [C]_L_ is the initial concentration of radioactivity in the donor chamber (in units of CPM/µL), [C]_C_ is the concentration of radioactivity in the receptor chamber (in units of CPM/µL) and Vc is the volume of the receptor chamber in μL. The volume cleared was plotted vs. time, and the slope was estimated by linear regression. The slopes of clearance curves for the iBEC monolayer plus Transwell^®^ membrane was denoted by PS_app_, where PS is the permeability × surface area product (in µL/min). The slope of the clearance curve for a Transwell^®^ membrane without iBECs was denoted by PS_membrane_. The PS value for the iBEC monolayer (PS_e_) was calculated from 1/PS_app_ = 1/PS_membrane_ + 1/PS_e_. The PS_e_ values were divided by the surface area of the Transwell^®^ inserts (0.33 cm^2^) to generate the endothelial permeability coefficient (Pe, in µL/min/cm^2^).

### Sodium fluorescein (NaF) permeability

The luminal and abluminal HESFM + 1% B27 mediums were refreshed, and cells were equilibrated in the incubator for 20 min. The 100 µL luminal (donor) chamber volume was switched to HESFM + 1% B27 + 100 µM NaF to initiate the assay, and 500 µL volumes of medium from the abluminal (receptor) chamber were collected and replaced with fresh, pre-warmed HESFM + 1% B27 medium after incubation times of 10, 20, 30, 60, and 120 min. Fluorescence was measured on a BioTek microplate reader and the concentration of NaF was calculated from a standard curve. The rate of accumulation, measured by plotting the accumulated NaF concentration vs. time, was corrected for flux across an empty transwell with no cells and used to calculate the permeability surface area coefficients (Pe), as described for glucose uptake assays.

### TNF-α treatments

Recombinant human tumor necrosis factor (TNF)-α (R&D Systems, cat no. 210-TA) was obtained as a lyophilized powder and stored at − 20 °C in a glass vial. Stock solutions (100 µg/mL) were prepared by reconstituting in sterile PBS and were stored in 25 µL aliquots at − 70 °C. Stock solutions were diluted in the existing luminal and abluminal mediums for 100 ng/mL TNF-α treatments. After 16 h, TEER was measured.

### Derivation of neurons from hiPSCs and conditioned medium treatments

We used previously established gene-edited hiPSC lines harboring the amyloid precursor protein (APP) Swedish mutation (Swe) and as well as the isogenic control described in Young et al. [25]. This mutation corresponds to a 2 bp change, GA to TC, in exon 16 of *APP*. We used cells heterozygous for this mutation, APP^Swe/+^, along with the isogenic APP wild-type, APP^WT^. Neurons from these cell lines were differentiated from hiPSCs as previously described in Knupp et al. [26] and D’Souza et al. [27]. hiPSC-neurons from the APP^Swe/+^ and APP^WT^ lines were cultured in Neural Differentiation media (1:1 DMEM/F12 (Life Technologies, cat no. 11039047) + neurobasal media (GIBCO, cat no. 21103049), 0.5% N2 supplement (Thermo Fisher Scientific, cat no. 17502-048) 1% B27 supplement (Thermo Fisher Scientific, cat no. 17504-044), 0.5% GlutaMax (Thermo Fisher Scientific, cat no. 35050061), 0.5% insulin-transferrin-selenium (Thermo Fisher Scientific, cat no. 41400045), 0.5% NEAA (Thermo Fisher Scientific, cat no. 11140050), 0.2% β-mercaptoethanol (Life Technologies, cat no. 21985023), 0.2 μg/mL brain-derived neurotrophic factor (Eurotech, cat no. 450–02), 0.2 µg/mL glial-cell-derived neurotrophic factor (PeproTech, cat no. 450-10), and 0.5 M dbcAMP (Sigma Aldrich, cat no. D0260) and cultured on Matrigel (Corning, cat no. 356231) at 37 °C in a 5% CO_2_ incubator. After 21 days of differentiation in Neural Differentiation media, conditioned medium (CM) was collected from each cell line following 72 h of contact with the mixed neuronal cell population. Aβ peptide content in CM was quantified using an Aβ Triplex ELISA plate (Meso Scale Discovery, cat no. 151200E-2). For iBEC conditioned medium (CM) treatments, transwells were distributed among groups (APP^WT^ or APP^Swe/+^) such that mean TEER values were approximately equal. The abluminal medium was replaced with CM collected from APP^WT^ or APP^Swe/+^ neuronal cultures. After 24 h, TEER was measured.

### Statistics

The Prism 9.0 statistical software package was used for all statistical calculations (GraphPad Inc, San Diego, CA, USA). Means are reported with their standard error (SE). For all figures, means are displayed with their SE and n. Linear regression lines and their slopes and intercepts were calculated using the Prism 9.0 software. Unpaired two-tailed t-tests were used to compare two means and analysis of variance (ANOVA) followed by Tukey’s multiple comparisons test when more than two means were compared.

## Results

### iBEC proliferation rate decreases over time spent in culture

**Fig. 1:**
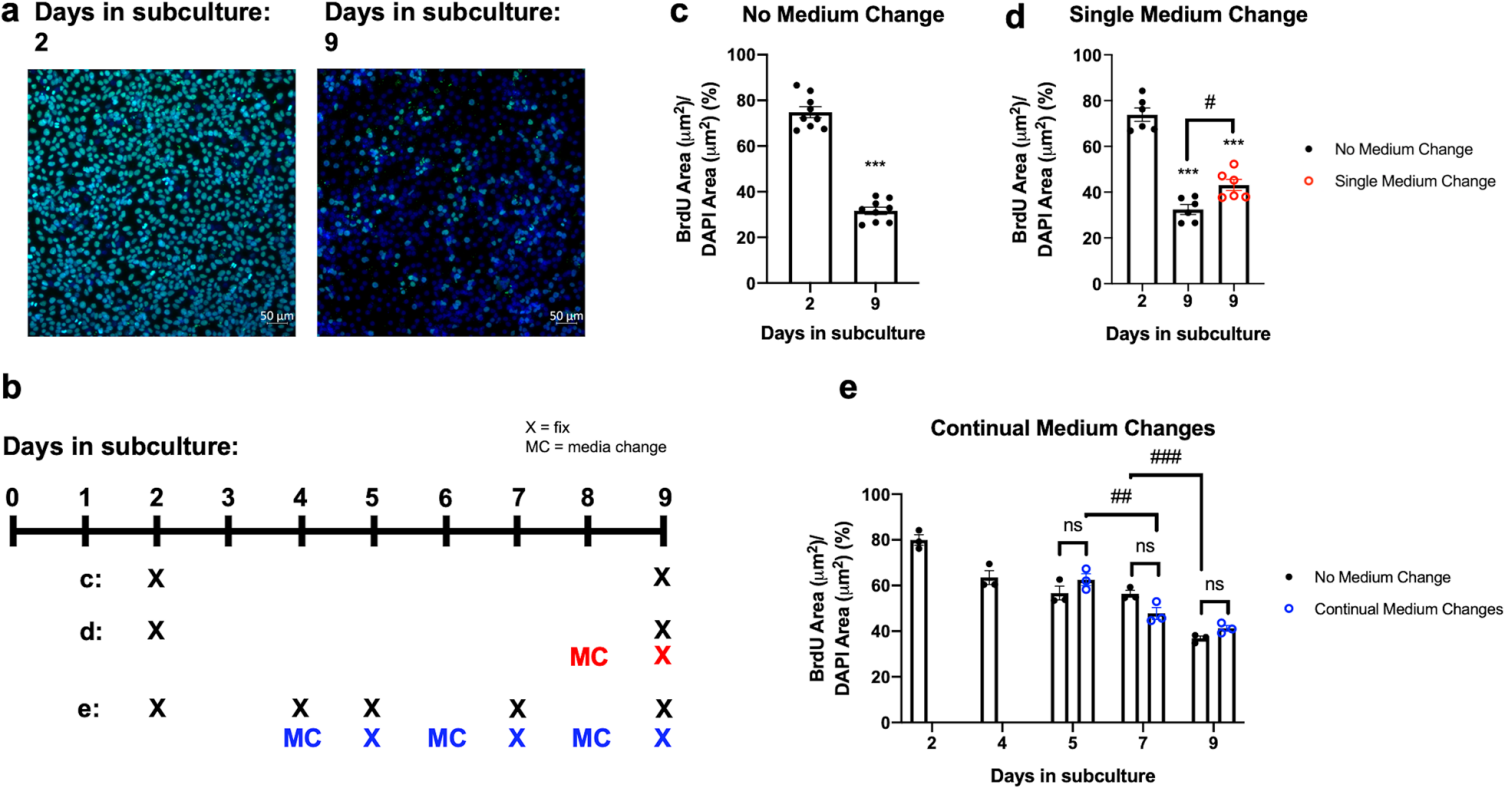
Effects of medium changes on BrdU incorporation in GM25256 iBECs. **a** Representative immunocytochemistry of BrdU (green) incorporation in iBECs on days 2 and 9 after subculture, counterstained with DAPI (blue). **b** Schematic depicting days of iBEC fixation (X) or media change (MC) corresponding to graphs c–e. **c** Immunofluorescence analysis of BrdU + area/DAPI + area in iBECs with no MC on days 2 and 9 after subculture. Each data point represents the average of 3–4 random fields of view per well. Three independent differentiations were performed with n = 3 wells imaged per group per differentiation. ***p < 0.001 (Unpaired two-tailed t-test). **d** Immunofluorescence analysis of BrdU + area/DAPI + area in iBECs days 2 and 9 after subculture with no MC vs. on day 9 with a single MC on day 8. Each data point represents the average of 3–4 random fields of view per well. Two independent differentiations were performed with n = 3 wells imaged per group per differentiation. #p < 0.05, ***p < 0.001 (vs. day 2) (One-way ANOVA with Tukey’s multiple comparisons test). **e** Immunofluorescence analysis of BrdU + area/DAPI + area in iBECs on days 2, 4, 5, 7 and 9 after subculture with no MC vs. on days 5, 7, and 9 with continual MC on days 4, 6, and 8 (day 5: single MC on day 4; day 7: two MC on days 4 and 6; day 9: three MC on days 4, 6, and 8. Each data point represents the average of 3 random fields of view per well. Not significant (ns), ##p < 0.01, ###p < 0.001 (Two-way ANOVA with Tukey’s multiple comparisons test used to compare no media change vs. continual media change groups on days 5, 7, and 9). **c–e** Means are displayed with their SE

In the adult vasculature, contact-inhibited endothelial cells (ECs) remain mostly quiescent [28]. In vitro studies using primary human ECs have shown that ECs adopt a quiescent state upon contact inhibition, which is associated with altered regulation of glucose metabolism and transport [29]. We hypothesized that iBECs would mature into a more quiescent state over an extended period of time in culture. To test this hypothesis, we assessed the DNA incorporation of 5-bromo-2'-deoxyuridine (BrdU) to determine the proportion of dividing cells at early (2 days) and late (9 days) post-subculture time points. We found that, over time, there was a significant decrease in the number of proliferating iBECs. (Fig. 1a–c). The percentage of BrdU positive cells was significantly lower in iBECs on day 9 post-subculture (31.63 ± 1.59%) relative to day 2 (74.80% ± 2.41%), corresponding to a 57.71% reduction in proliferation over one week in standard culture conditions with no medium changes (NMC). Further, we found that the reduction in proliferation is accompanied by a significant decrease in cell density (Additional file 1: Fig. S1). To determine whether nutrient replenishment can restore iBEC proliferation, we tested the effects of a single medium change (SMC) on the extent of BrdU incorporation (Fig. 1b). SMC on day 8 post-subculture induced a modest increase in iBEC proliferation on day 9 compared to NMC (32.37% ± 2.13% vs. 43.05% ± 2.46%, respectively) (Fig. 1d). We also tested whether re-application of the pro-angiogenic effectors basic fibroblast growth factor (bFGF) and retinoic acid (RA) on day 8 could stimulate iBEC proliferation and found no change in BrdU incorporation on day 9 in response to bFGF/RA treatment (Additional file 2: Fig. S2). Given the slight, but significant, effect of SMC on iBEC proliferation on day 9, we aimed to determine whether the decline in iBEC proliferation occurs dependently or independently of nutrient depletion in the medium over time. We performed continual medium changes (CMC) of both the luminal and abluminal mediums on days 4, 6 and 8 post-subculture, and examined the effects on BrdU incorporation (Fig. 1b). The proliferative status of iBECs on day 9 with NMC was not significantly different than with CMC (Fig. 1e). In fact, no significant differences between the NMC and CMC groups were detected on any day. Two-way ANOVA comparing the NMC vs. CMC groups on days 5, 7, and 9 revealed a significant (***p < 0.001) main effect of day in subculture accounting for 78.39% of the total variation, while the effects of medium changes accounted for only 0.11%. While the decline in proliferation from day 2 to day 9 post-subculture is marginally prevented with nutrient replenishment on day 8, recurring nutrient replenishment does not significantly affect iBEC proliferation, demonstrating that iBEC adoption of a more quiescent state is only minimally influenced by diminishing nutrient availability in the medium over time.

### Tight and adherens junction protein expression changes and TEER as iBEC proliferation declines

**Fig. 2:**
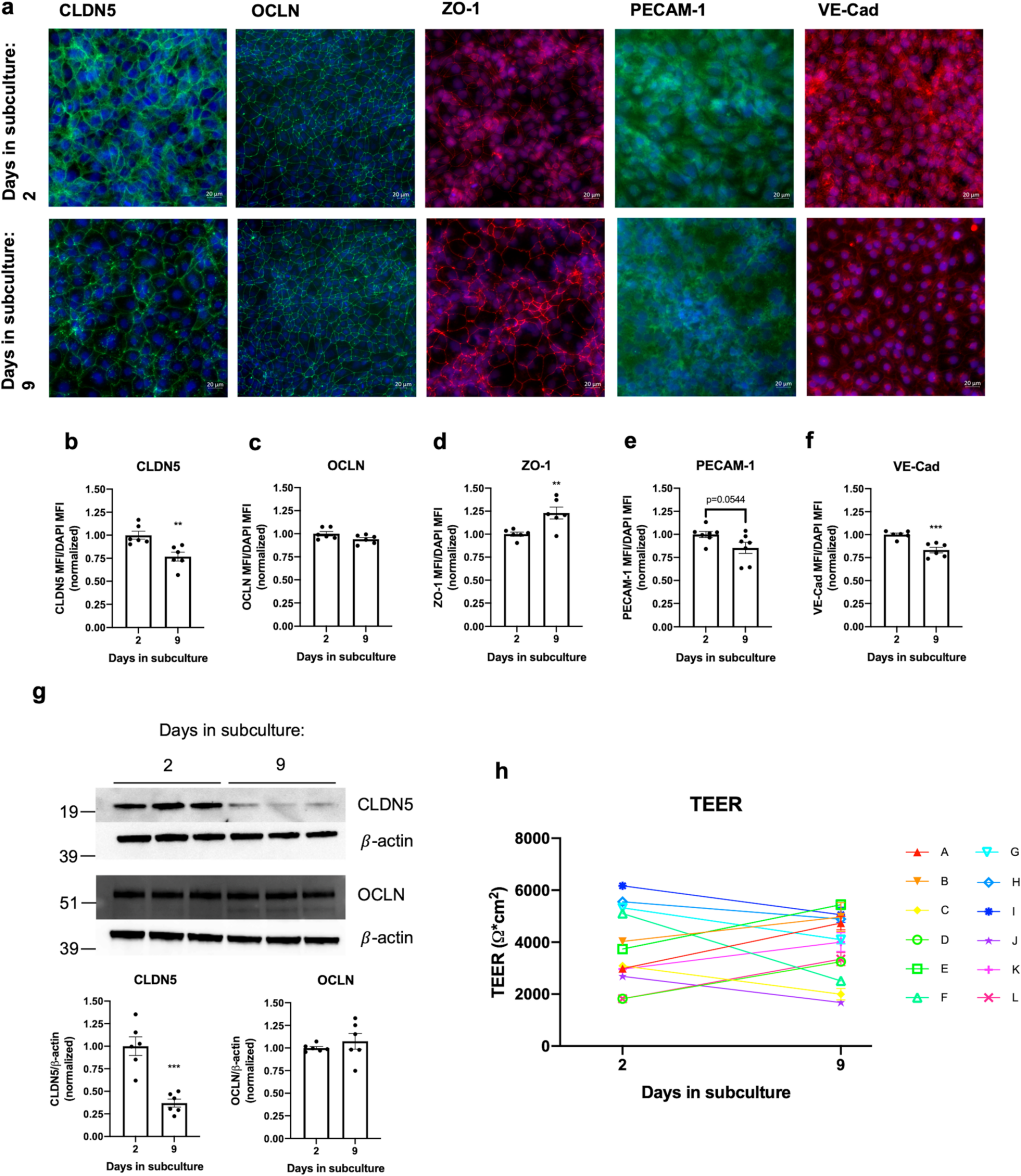
Immunocytochemistry and immunofluorescent analyses of TJP and AJP in GM25256 iBECs. **a** Representative immunocytochemistry of CLDN5 (green), OCLN (green), ZO-1 (red), PECAM-1 (green), and VE-Cad (red) on days 2 and 9 after subculture, counterstained with DAPI (blue). **b–f** Immunofluorescence analyses of CLDN5, OCLN, ZO-1, PECAM-1, and VE-Cad mean fluorescence intensities (MFI) relative to DAPI MFI on days 2 and 9 after subculture (normalized to day 2). Each data point represents the average of 3–4 random fields of view per well. Two independent differentiations were performed with n = 3 wells imaged per differentiation. **p < 0.01, ***p < 0.001 (Unpaired two-tailed t-test). Means are displayed with their SE. **g** Representative immunoblots and immunoblot quantification of CLDN5 and OCLN expression on days 2 and 9 after subculture. Expression was calculated relative to $\beta$-actin and normalized to day 2. Each data point represents one well. Two independent differentiations were performed with n = 3 wells for each differentiation. ***p < 0.001 (Unpaired two-tailed t-test). Means are displayed with their SE. **h** TEER trajectories of GM25256 iBECs. TEER was evaluated on days 2 and 9 after subculture. Each line represents an independent differentiation of iBECs (n > 10 for all differentiations). Means of technical replicates are displayed

We next aimed to characterize changes in TJP and AJP expression in relation to TEER in proliferative vs. more quiescent iBECs. All measurements were normalized to DAPI to correct for reduction in cell density between days 2 and 9. Immunofluorescence analyses (IFA) comparing TJP expression on day 2 vs. day 9 post-subculture indicated a significant decrease in claudin-5 (CLDN5) expression while occludin (OCLN) expression remained unchanged and zonula occludens-1 (ZO-1) expression increased (Fig. 2a, b–d). Further, we detected a significant decrease in expression of the AJP vascular endothelial-cadherin (VE-Cad), while there was a trend towards a decrease in platelet endothelial cell adhesion molecule-1 (PECAM-1) expression (p = 0.0544) (Fig. 2a, e–f). Immunoblot analyses of CLDN5 and OCLN expression confirmed the results of the IFA (Fig. 2g). Qualitative differences in TJP and AJP localization were also noted between days 2 and 9 (Fig. 2a), with CLDN5, ZO-1, PECAM-1 and VE-Cad appearing to be more localized to the junctions on day 9. It is possible that the more diffuse staining of these proteins in iBECs’ more proliferative state reflects increased intracellular trafficking/storage to support high rates of mobilization to the junctional zones during a period of heightened protein turnover, and conversely, that reduced proliferation allows for protein complex stabilization, resulting in enhanced junctional localization in more quiescent iBEC monolayers. Though changes in junctional protein expression are commonly associated with changes in barrier functions, we found that TEER trajectories from day 2 to day 9 post-subculture did not show consistent patterns of change, and that there was no difference of mean TEER on day 2 vs. on day 9. (Fig. 2h). Therefore, the alterations in TJP/AJP expression and localization could reflect mechanisms that preserve TEER as the iBECs transition to a more quiescent state, and/or as cells in the monolayer are lost. Importantly, the high TEER values of iBECs on day 9 suggest that the reduced expression of the key junctional proteins CLDN5 and VE-Cad is not detrimental to junctional integrity in our model. Because strong barrier functions are maintained with prolonged culture, our findings support the use of this model to study junctional destabilization in both proliferative and quiescent states.

### iBEC expression patterns reflect the endothelial transition to a more quiescent metabolic state

**Fig. 3:**
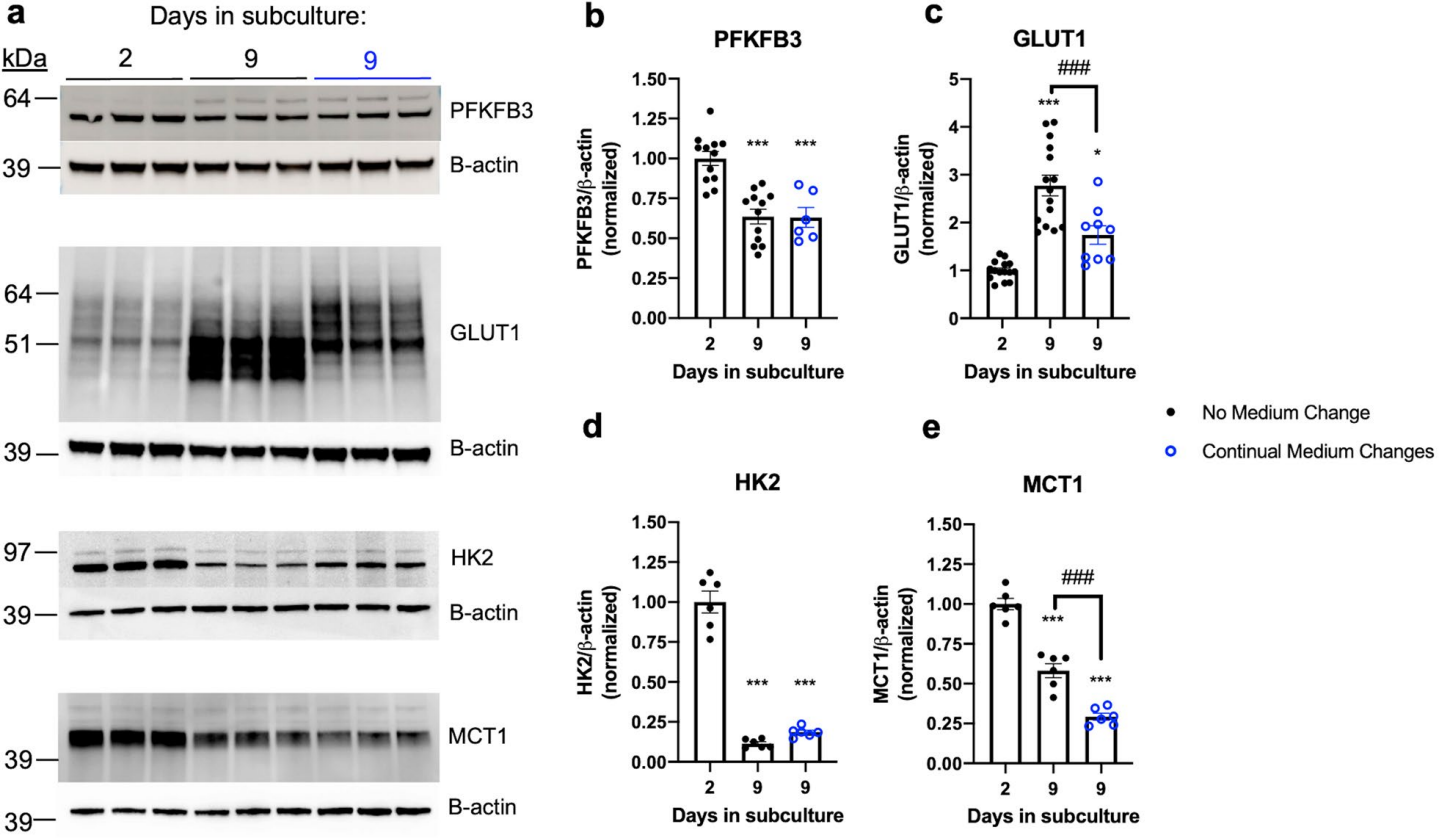
Effects of length of subculture and medium changes (MC) on iBEC expression of glucose metabolism/transport-related proteins. **a** Representative immunoblots of PFKFB3, GLUT1, HK2, and MCT1 expression on days 2 and 9 after subculture with no MC (black) vs. on day 9 after continual MC on days 4, 6, and 8 (blue). **b–e** Quantification of immunoblots. Expression was calculated relative to $\beta$-actin and normalized to day 2. Each data point represents one well. For PFKFB3 and GLUT1, two independent differentiations were performed with n = 3 wells per group to evaluate expression of days 2 and 9 after subculture with no media changes. Then, two more independent differentiations were performed with n = 3 wells per group to additionally evaluate expression on day 9 with continual media changes on days 4, 6, and 8. For HK2 and MCT1, two independent differentiations were performed which evaluated all three groups with n = 3 wells per group. *p < 0.05, ***p < 0.001 (vs. day 2); ###p < 0.001 (One-way ANOVA with Tukey’s multiple comparisons test). Means are displayed with their SE

ECs predominantly derive ATP from glucose metabolism to lactate through glycolysis, rather than oxidative phosphorylation [30]. As ECs become quiescent, they adapt to lower metabolic requirements by repressing their glycolytic activity, characterized by downregulation of 6-phosphofructo-2-kinase/fructose-2,6-biphosphatase 3 (PFKFB3), which produces large amounts of fructose-2,6-bisphosphate to activate phosphofructokinase-1 (PFK1), a rate-limiting enzyme in glycolysis. Upregulation of GLUT1 is concomitant with the reduction in glycolysis, suggesting that the majority of glucose entering via GLUT1 is transported across the quiescent endothelium, while only a minor fraction is used for energy metabolism [29, 31]. Therefore, we aimed to determine whether iBECs adopt similar expression changes to primary ECs during the transition to a more quiescent state, and the effects of CMC. We found that, with NMC, iBEC expression of PFKFB3 was significantly reduced on day 9 relative to day 2, while GLUT1 expression was significantly increased (Fig. 3a–c). PFKFB3 expression on day 9 with CMC was similar to that with NMC. However, GLUT1 expression on day 9 was significantly lower with CMC than with NMC, suggesting that GLUT1 upregulation may be, in part, enhanced by glucose depletion. Furthermore, we observed a greater abundance of lower molecular weight (MW) GLUT1 isoforms with NMC, while higher MW isoforms predominated with CMC. As glucose is a primary building block for glycosylation, this finding is consistent with predominance of more glycosylated GLUT1 isoforms when glucose is replete in the medium. We compared the GLUT1 MW profile of iBECs on day 2 and day 9 with that of primary human astrocytes. We found that iBECs on day 2 expressed higher MW isoforms than primary human astrocytes, consistent with the higher extent of glycosylation on brain endothelial GLUT1 compared to astrocytic GLUT1 [16]. In comparison, iBECs on day 9 expressed both the higher isoforms of day 2 iBECs and the lower MW isoforms of primary human astrocytes (Additional file 3: Fig. S3). In addition, we evaluated expression changes of hexokinase 2 (HK2), which catalyzes the first rate-limiting reaction of glycolysis by phosphorylating glucose [32], and monocarboxylate transporter-1 (MCT1), which facilitates the bidirectional transport of lactate, a metabolic waste product of glycolysis and a signaling agent that promotes angiogenesis [33]. We found HK2 expression was significantly decreased in iBECs on day 9 relative to day 2, which was maintained with CMC (Fig. 3a, d). MCT1 expression was also significantly reduced in iBECs from day 2 to day 9 (Fig. 3a, e). Interestingly, downregulation of MCT1 was enhanced by CMC. Overall, the glucose metabolism and transport-related protein expression changes exhibited by more quiescent iBECs reflect a systematic repression of glycolysis, recapitulating the phenotype of quiescent primary ECs.

### Transendothelial glucose transport system function is enhanced in proliferating iBECs

**Fig. 4:**
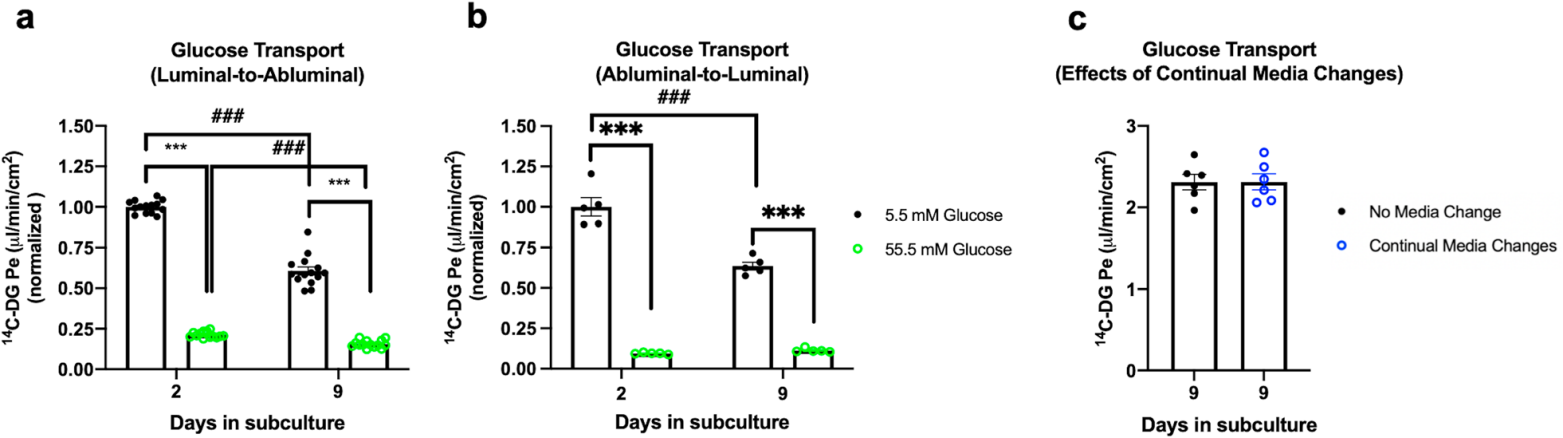
Glucose transport kinetics in GM25256 iBECs. **a** Comparison of luminal-to-abluminal glucose transport saturability kinetics on days 2 and 9 after subculture. Glucose transport rates (^14^C-DG Pe) were compared in the presence (55.5 mM Glucose, green) or absence (5.5 mM Glucose, black) of excess glucose in the luminal compartment to examine glucose transport system saturability. Three independent differentiations were performed to with n = 4–5 transwells per group. **b** Comparison of abluminal-to-luminal glucose transport saturability kinetics on days 2 and 9 after subculture. 14C-DG Pe values were compared in the presence (55.5 mM Glucose, green) or absence (5.5 mM Glucose, black) of excess glucose in the abluminal compartment to examine glucose transport system saturability. One differentiation was performed with n = 5 transwells per group. ***/###p < 0.001 (Two-way ANOVA with Tukey’s multiple comparisons test). **a–b** ^14^C-DG Pe values were normalized to the unsaturated Pe on day 2. **c** Luminal-to-abluminal ^14^C-DG Pe on day 9 after subculture were compared with (blue) and without (black) continual media changes on days 4, 6, and 8. One differentiation was performed with n = 6 transwells per group. (Unpaired two-tailed t-test). **a–c** Means are displayed with their SE

To determine whether the increased GLUT1 expression observed in more quiescent iBECs is accompanied by enhanced glucose transport kinetics, we assayed transendothelial transport of ^14^C-2-deoxy-d-glucose (^14^C-DG) across monolayers of iBECs on day 2 and day 9 post-subculture. As ^14^C-DG becomes trapped in the cell once it is phosphorylated by hexokinase, only unmetabolized ^14^C-DG is transported to exclusively proxy transendothelial glucose transport [23, 34]. The permeability-surface area coefficients (Pe) for luminal-to-abluminal and abluminal-to-luminal ^14^C-DG transport were measured in the presence and absence of excess glucose in the donor chamber (55.5 mM Glucose and 5.5 mM Glucose, respectively) to assess glucose transport system saturability. The Pe for ^99m^Tc-DTPA was assessed simultaneously to measure non-specific para- and transcellular leakage. Surprisingly, we found a significant 39.33% decrease in the unsaturated rate of luminal-to-abluminal ^14^C-DG transport in iBECs on day 9 relative to day 2 (Fig. 4a). No significant effect of either day in subculture or excess glucose on iBEC permeability to ^99m^Tc-DTPA was detected (Additional file 4: Fig. S4a). We found a similar 36.44% decrease in the unsaturated rate of abluminal-to-luminal ^14^C-DG transport from day 2 to day 9, evidencing a reduced capacity of the bidirectional glucose transport system in iBECs’ more quiescent state (Fig. 4b). In this experiment, a significant (***p < 0.001) main effect of day in subculture was detected where abluminal-to-luminal leakage of ^99m^Tc-DTPA was significantly increased on day 9 relative to day 2 (Additional file 4: Fig. S4b).

To compare leakage of ^99m^Tc-DTPA Pe to a more commonly used fluorescent leakage marker, we measured sodium fluorescein (NaF) Pe on day 2 post-subculture. The average TEER of iBECs used for this transport assay was 4360.17 ± 14.69 $\Omega$*cm^2^, and the average NaF Pe was 1.70*10^–2^ ± 6.30*10^–4^l$\mu$l/cm^2^/min, which corresponds to 2.83*10^–7^ ± 1.05*10^–8^ cm/s. The average ^99m^Tc-DTPA Pe for iBECs from the same differentiation on day 2 was 2.21*10^–3^ ± 4.24*10^–4^l$\mu$l/cm^2^/min. The NaF Pe in GM25256 iBECs is similar to that reported for iBECs derived from other hiPSC lines [14, 15] and is also similar to ^99m^Tc-DTPA Pe, supporting that ^99m^Tc-DTPA permeability approximates that of NaF.

Saturation with excess glucose in the donor compartment produced significant reductions in ^14^C-DG on both day 2 and day 9, demonstrating the iBEC glucose transport system is similarly saturable in both proliferative and more quiescent states. We confirmed that the significant decrease in ^14^C-DG Pe upon the addition of excess glucose was due to saturation of the glucose transport system rather than hyperosmotic stress by comparing the effect of 55.5 mM glucose vs. 55.5 mM mannitol. 55.5 mM mannitol did not significantly affect either ^14^C-DG Pe (Additional file 4: Fig. S4c) or ^99m^Tc-DTPA Pe (data not shown). In addition, we confirmed that ^14^C-DG Pe could be inhibited by the GLUT1-selective inhibitor BAY-876. 2 µM BAY-876 produced significant reductions in ^14^C-DG Pe on both day 2 and day 9 post-subculture (34.96% and 34.20%, respectively) (Additional file 4: Fig. S4d) and did not significantly affect ^99m^Tc-DTPA Pe on either day (data not shown). To test whether the rate of glucose transport is regulated by nutrient availability in the medium, we compared ^14^C-DG Pe on day 9 in iBECs with NMC vs. CMC. ^14^C-DG Pe was the same in both conditions (Fig. 4c), demonstrating that changes in nutrient availability likely do not mediate the difference in the glucose transport system functionality between proliferative and more quiescent iBECs. To quantitatively evaluate the bidirectional glucose transport system in iBECs, we compared luminal-to-abluminal and abluminal-to-luminal ^14^C-DG Pe in the same differentiation on day 2, where glucose transport in both directions was measured simultaneously. We found that the rate of luminal-to-abluminal glucose transport was significantly higher than the rate of abluminal-to-luminal glucose transport (Additional file 4: Fig. S4e), showing that the model supports a net flux of glucose in the blood-to-brain direction. In summary, our findings indicate that (1) while the expression level of GLUT1 is regulated by changes in nutrient availability, the rate of transendothelial glucose transport is not, and (2) increased GLUT1 expression as iBECs become more quiescent does not correspond with increased functional glucose transport.

### iBEC proliferation confers a selective vulnerability to an Alzheimer’s Brain Milieu, but not TNF-α

**Fig. 5:**
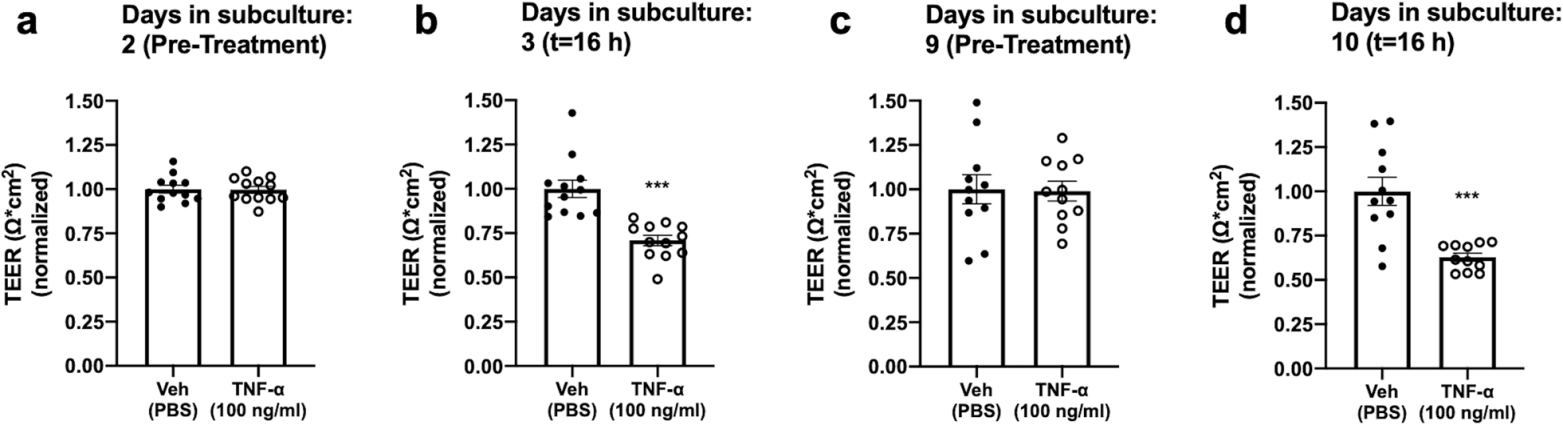
Effects of TNF-⍺ treatment on TEER. **a–d** On day 2 or day 9, transwells were organized into treatment groups such that TEER means were approximately equal and treated both luminally and abluminally with 100 ng/ml TNF-⍺ or PBS vehicle (Veh) control**.** TEER was measured after 16 h treatment, on day 3 or day 10, respectively. Two independent differentiations of iBECs (Exp I and II) were performed with n = 5–7 transwells per group. TEER values were normalized to Veh control. Values used to normalize results (in $\Omega$*cm^2^) are 3315.64 (Exp I Day 2), 3946.00 (Exp II Day 2), 2956.04 (Exp I Day 3), 2634.52 (Exp II Day 3), 4089.16 (Exp I Day 9), 2859.42 (Exp II Day 9), 4546.61 (Exp I Day 10), 3291.39 (Exp II Day 10). ***p < 0.001 (Unpaired two-tailed t-test). Means are displayed with their SE

**Fig. 6:**
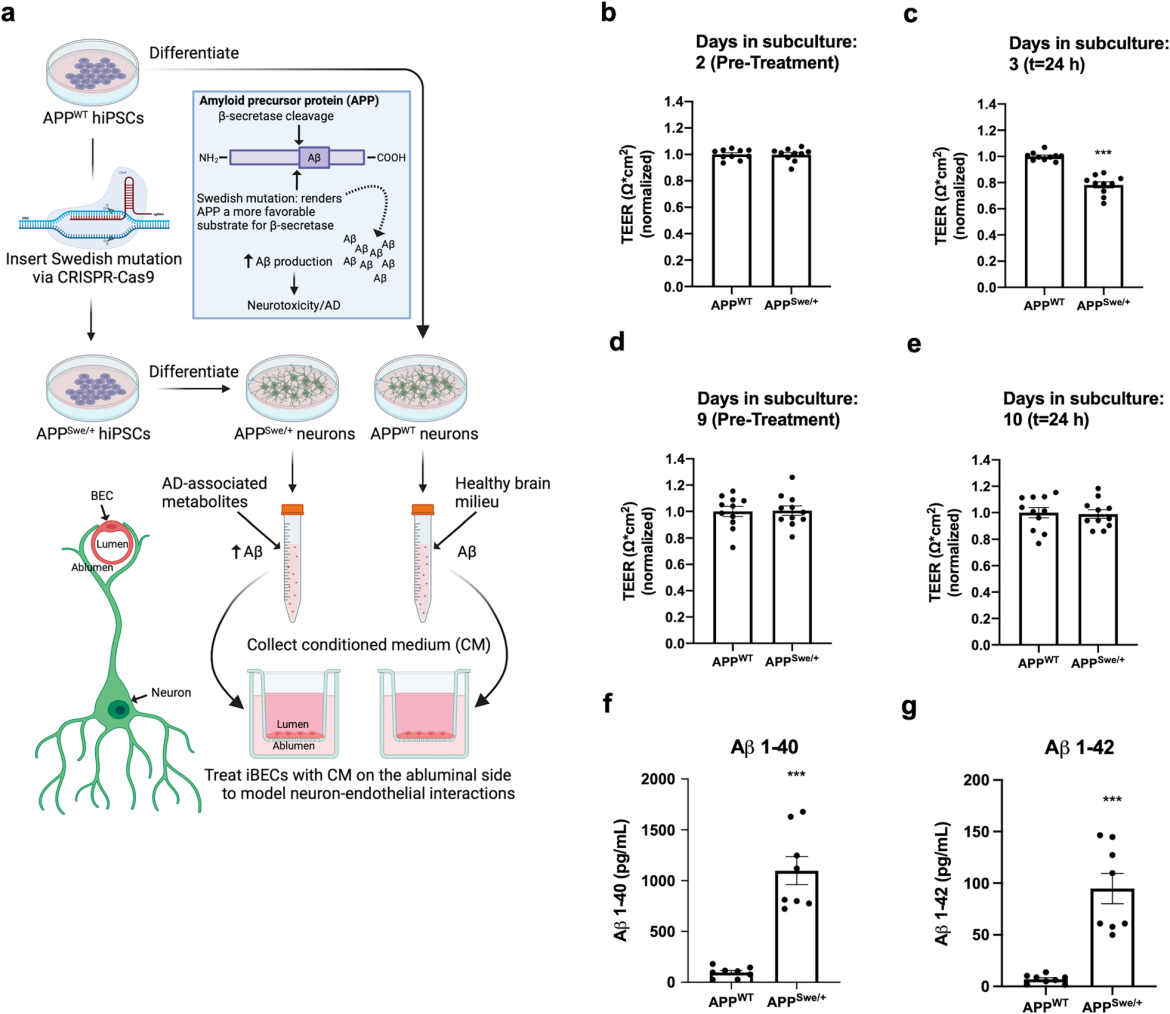
Modeling neuron-endothelial interactions in Alzheimer’s disease using iBECs treated with conditioned media (CM) from hiPSC-derived neurons. **a** Schematic depicting CM treatments. Created with Biorender.com. **b–e** On day 2 or day 9, transwells were organized into treatment groups (APP^WT^ and APP^Swe/+^) such that TEER means were approximately equal and treated with hiPSC-neuronal CM. The CM effect on iBEC TEER was measured after 24 h treatment, on day 3 or day 10, respectively. Two independent differentiations of iBECs (Exp I and II) were conducted to test conditioned media from one differentiation of hiPSC-neurons (n = 3–4 transwells per group). Results were confirmed with one additional differentiation of iBECs (Exp III) treated with CM from another differentiation of hiPSC-neurons (n = 4–5 transwells per group). TEER values were normalized to APP^WT^ control. Values used to normalize results (in $\Omega$*cm^2^) are 5228.93 (Exp I Day 2), 4134.45 (Exp II Day 2), 5837.78 (Exp III Day 2), 3967.91 (Exp I Day 3), 2888.06 (Exp II Day 3), 4544.19 (Exp III Day 3), 2567.82 (Exp I Day 9), 5497.35 (Exp II Day 9), 4288.35 (Exp III Day 9), 1989.64 (Exp I Day 10), 3448.05 (Exp II Day 10), 3709.38 (Exp III Day 10). ***p < 0.001 (Unpaired two-tailed t-test). **f–g** Quantification of A $\beta$-40 and -42 in APP^WT^ and APP^Swe/+^ conditioned mediums (CM). Four independent differentiations of hiPSC-neurons were performed with n = 2 wells quantified per differentiation. **b–g** Means are displayed with their SE

We next determined whether the proliferative state of iBECs affects their vulnerability to an inflammatory stimulus. We compared the effects of 100 ng/mL TNF-α treatment (applied to both luminal and abluminal compartments) on day 2 vs. day 9 post-subculture. We found significant decreases in TEER after treatment on both days (29.12% and 37.17%, respectively), indicating that iBEC vulnerability to TNF-α-induced BBB disruption is not dependent on iBEC proliferative status (Fig. 5a–d). Finally, we investigated whether an Alzheimer’s disease (AD)-associated stimulus also produced comparable disruption in proliferative and more quiescent iBEC states. We modeled neuron-endothelial interactions in AD by treating iBECs on the abluminal side with conditioned medium (CM) collected from neuronal cultures derived from hiPSCs harboring one copy of the Swedish mutation (APP^Swe/+^) (Fig. 6a). This familial AD-causing mutation renders the amyloid precursor protein (APP) a more favorable substrate for cleavage by β-secretase (BACE-1), causing increased production of amyloid beta (A $\beta$) [35]. We compared this with CM collected from hiPSC-neurons derived from the isogenic control line, APP^WT^, which does not harbor the Swedish mutation. Based on previously published reports indicating enhanced susceptibility of proliferating ECs to A $\beta$ [36], we predicted that greater reductions in TEER would be induced by APP^Swe/+^-CM treatment on day 2 vs. on day 9. On day 2, treatment of iBECs with APP^Swe/+^-CM produced a significant decrease in TEER compared with control CM from the APP^WT^-derived hiPSC-neurons (Fig. 6b, c). In contrast, we did not observe a significant decrease in TEER relative to Cvi-A2 control following APP^Swe/−^-CM treatment on day 9 (Fig. 6d–e). Our data suggest that the higher rate of iBEC proliferation on day 2 confers a selective vulnerability to paracellular disruption caused by an Alzheimer’s brain-like milieu. Though APP^Swe/+^-CM contains elevated A $\beta$-40 and -42 levels compared to APP^WT^ [25] (Fig. 6f, g), future studies are needed to elucidate whether the effects of APP^Swe/+^-CM are A $\beta$-mediated.

## Discussion

A unique feature of the iBEC model is its ability to maintain high TEER over a sustained period of time in culture. The maintenance of barrier properties suggests that iBECs are amenable to longer in vitro studies, which could aid the understanding of human disease mechanisms. However, little is currently known about the stability of the iBEC phenotype with prolonged culture. As we demonstrate here, GM25256 iBECs become more quiescent over time accompanied by adoption of a metabolic phenotype that is similar to primary ECs cultured to contact-inhibition [29]. A limitation of the current study is that we do not know whether iBECs from other commonly used iPSC lines adopt a similar quiescent phenotype, but our work is a first step in showing that GM25256-derived iBECs are suitable for studies on quiescence-related changes to BBB functions.

We first posited that factors inherent to the culture method such as nutrient availability and/or the time elapsed from bFGF and RA exposure could potentially regulate the proliferative status of iBECs [37]. However, we found that continual medium changes (CMC) could not prevent the transition to a more quiescent state that occurs from day 2 to day 9 post-subculture, nor could the re-application of bFGF/RA fully restore the proliferative status of iBECs after prolonged culture. Only a modest increase in proliferative cells was observed when medium was changed on day 8 post-subculture. These findings indicate that the growth arrest that occurs in iBECs is stable and occurs independently of nutrient depletion or the absence of bFGF and RA.

We and others have consistently observed the preservation of high TEER in iBECs over extended periods of time in subculture [14, 15]. Here, we demonstrate that TEER is not dependent on length of subculture, as strong barrier functions were upheld in both proliferative and more quiescent iBECs states and there was no mean difference in TEER between early and late post-subculture time points. However, the expression of some TJP and AJP was dependent on length of subculture. Firstly, we found a significant loss of CLDN5 expression over time. Previous studies have also linked increased CLDN5 expression with a more angiogenic EC state [38]. Interestingly, CLDN5 overexpression promoted proliferation in hCMEC/D3 cells (immortalized human BEC line), while silencing it blocked cell cycle progression at the G0/G1 phase [39]. Although decreased CLDN5 levels are commonly associated with barrier disruption [40], upregulation of CLDN5 has also been seen in the context of diminished TEER and increased paracellular permeability [11, 41]. Intense CLDN5 staining was observed at the junctional zones of more quiescent iBEC monolayers, supporting that, despite reduced expression, CLDN5 expression is sufficient and appropriately localized to support the maintenance of high TEER. In agreement with existing literature, we found ZO-1 expression is significantly increased in more quiescent iBECs. Reduced expression of ZO-1 generally correlates with increased cell proliferation, such as in highly proliferative BECs from human brain tumors [42, 43]. ZO-1 has been reported to accumulate in the nucleus of proliferating epithelial cells [44], whereas in high density confluent cells, ZO-1 is primarily localized to cell–cell junctions and inhibits cell proliferation by indirectly controlling expression of cell cycle regulators [44, 45]. Our immunofluorescence analyses yielded clear evidence of enhanced ZO-1 junctional localization in more quiescent iBECs. We found OCLN levels did not change significantly as proliferation declined, indicating regulation of OCLN expression is likely not modulated by iBEC proliferative state.

For the AJPs evaluated, PECAM-1 expression was not significantly altered over time spent in subculture. However, we found a significant reduction in VE-cadherin expression. VE-cadherin is important in contact inhibition of proliferation [46]. Although its expression was decreased over time, VE-cadherin junctional localization was maintained at day 9 post-subculture, raising the possibility of its involvement in iBEC adoption of quiescence. Taken together, our data underscore that changes in the levels of any one TJP do not necessarily translate to changes in barrier tightness. This concept has been previously supported in studies demonstrating modulations of BBB permeability with no changes in protein levels [47–49]. How junctional remodeling in highly proliferative BECs occurs without loss of barrier integrity remains to be fully elucidated.

Prior works have characterized iBECs as primarily utilizing glycolysis vs. oxidative phosphorylation for ATP synthesis [9], which aligns with the metabolic phenotype of primary ECs [30, 50]. Here, we demonstrate that iBECs undergo a metabolic transition from day 2 to day 9 post-subculture that resembles that of quiescent primary ECs, which alter their metabolism from the high energy requirements of proliferation to the lower energy requirements of quiescent barrier maintenance by downregulating glycolysis [29, 31]. The distinctive expression changes induced in primary ECs upon contact-inhibition, namely, downregulation of PFKFB3 and upregulation of GLUT1, also occur in iBECs during the transition from a highly proliferative state on day 2 post-subculture to a more quiescent state on day 9 post-subculture. Importantly, we found that nutrient replenishment with CMC did not alter the expression of PFKFB3. Nutrient replenishment did significantly suppress the increase in GLUT1 expression, indicating that GLUT1 expression is regulated by nutrient availability in addition to the proliferative status of iBECs. This finding is consistent with increased BBB GLUT1 expression in response to hypoglycemia [51].

We further identified reduced HK2 expression and reduced MCT1 expression as two additional indicators of glycolytic repression in iBECs. HK2 is necessary to support EC proliferation as it catalyzes the first rate-limiting reaction in glycolysis, and its depletion inhibits glycolysis and impairs angiogenesis [32, 52]. Like PFKFB3, HK2 downregulation over time was not affected by CMC. MCT1 facilitates the bidirectional transport of lactate. As MCT1 expression is induced by lactate [53], decreased lactate production via glycolysis likely contributes the reduced MCT1 expression in more quiescent iBECs on day 9. Furthermore, lactate has been shown to function as a pro-angiogenic effector to promote proliferation in BECs [54]. Therefore, downregulation of MCT1 may also be a mechanism to inhibit iBEC proliferation by decreasing lactate influx. CMC further suppressed the expression of MCT1, which may be attributed to removal of the lactate produced by the iBECs during glycolysis. Overall, our findings show that more quiescent iBECs adopt a metabolic phenotype reminiscent of quiescent primary ECs. Our results highlight the utility of the model as a platform to study the regulation of BBB functions under defined proliferative and metabolic states.

We confirmed a previous report of a functional and saturable iBEC glucose transport system [54] and that a GLUT1-selective inhibitor, BAY-876, significantly inhibits glucose transport in GM25256 iBECs. Endothelial upregulation of GLUT1 that occurs concurrently with quiescence is considered to be a mechanism of prioritizing glucose delivery to the surrounding tissues by more mature cells [29]. Therefore, we predicted that more quiescent iBECs, which were shown to have higher GLUT1 protein levels, would facilitate higher rates of glucose transport. However, the rate of glucose transport, as measured by ^14^C-DG Pe, was significantly lower in more quiescent iBECs vs. proliferative iBECs, which could not be explained by differences in leakage as quantified by ^99m^Tc-DTPA Pe. Further, the glucose transport rate of iBECs on day 9 post-subculture was not significantly altered by CMC, showing that nutrient depletion does not contribute to decreased glucose transport in more quiescent iBECs.

We also found that luminal-to-abluminal ^14^C-DG Pe was significantly higher than abluminal-to-luminal ^14^C-DG Pe, which suggests that the net flux of glucose across iBEC monolayers occurs in the blood-to-brain direction. The localization of GLUT1 on the luminal vs. abluminal membrane is one mechanism by which BECs may regulate the rate of glucose transport. In animal models, the luminal: abluminal ratio of GLUT1 at the BBB has been reported as 1:4 [55, 56], whereas in humans, the ratio was measured as 1:1 [57, 58]. Importantly, the transcytosis of glucose in either direction should be limited by the membrane with the fewest functional glucose transporters. We observed a decrease in the transport of ^14^C-DG in both directions on day 9, which corresponded with an increased expression of GLUT1 and a change in its MW. This discrepancy could be explained by enhanced asymmetry or localization of GLUT1 in a subcellular compartment other than the plasma membrane. It has been shown that GLUT1 translocation to the plasma membrane is one mode of glucose transport upregulation, which can occur without changes in GLUT1 protein expression [59]. Further, it has been shown that up to 40% of GLUT1 is sequestered in the cytoplasm of BECs [60].

^14^C-DG Pe is analogous to fluorodeoxyglucose (FDG), which is an ^18^F-labeled PET ligand that is routinely used to evaluate glucose uptake into tissues, including the brain [61]. Decreased brain uptake of FDG can be indicative of impaired glucose utilization by neurons, but also GLUT1 dysfunction at the BBB [62, 63]. An advantage of using ^14^C-DG as a glucose surrogate is that only unmetabolized ^14^C-DG can fully traverse the iBEC monolayer since it becomes trapped within the cell following phosphorylation to 2-deoxyglucose-6-phosphate by hexokinase, and this form is unable to be further metabolized by glycolytic enzymes [23, 34]. Although our assays did not have the sensitivity to detect intracellular 2-deoxyglucose-6-phosphate accumulation, we posit that the reduced transport ^14^C-DG in more quiescent iBECs would not be due to increased metabolism, since glycolytic enzymes, including HK2, are reduced.

In addition to observing differences in the total amount of GLUT1, we also observed the appearance of lower MW isoforms of GLUT1 in more quiescent iBECs, suggesting differences in post-translational modifications (PTM). Glycosylation is one PTM which alters the MW of GLUT1 [64], and can also regulate its function by facilitating membrane clustering on lipid rafts [65]. However, some of the apparent shifts in GLUT1 MW were mitigated by medium changes, which had no effect on functional glucose transport in our studies. It is also possible that other PTMs such as phosphorylation could regulate GLUT1 activity [66]. Presently, very little is known about the mechanisms of GLUT1 regulation in proliferating vs. quiescent BECs. iBECs could potentially be used to delineate these mechanisms as a future direction.

Prior studies have shown that iBECs are responsive to pro-inflammatory cytokine stimulation within two days post-subculture [67]. We explored the functional consequences of altered proliferation by comparing iBEC vulnerability to barrier disrupting insults on day 2 vs. day 9. TNF-α treatment produced comparable disruption in proliferative and more quiescent iBEC states. Therefore, the effects of TNF-α on TEER appear to be independent of the proliferative status of iBECs. Additionally, we modeled an Alzheimer’s brain milieu using conditioned medium (CM) from neuronal cultures differentiated from hiPSCs harboring the Swedish mutation, a familial AD-causing mutation resulting in elevated A $\beta$ production [35]. We found that in proliferative iBECs, CM from hiPSC-neurons harboring the Swedish mutation (APP^Swe/+^) caused a significant reduction in TEER, whereas CM from isogenic control hiPSC-neurons (APP^WT^) did not change TEER. In contrast, CM from neurons derived from either hiPSC line did not have a significant effect on TEER in more quiescent iBECs. These data suggest that BBB disruption in response to this AD-associated stimulus is dependent on iBEC proliferative state.

Our results align with a previous study showing a selective vulnerability of proliferative aortic ECs to the injurious effects of A $\beta$ [36] and suggest that actively dividing BECs, such as those in injured or growth states, are more vulnerable to disruption by Aβ-associated metabolites. In vitro A $\beta$ treatment studies generally require concentrations in to nM to µM range to induce BBB disruption [68], whereas A $\beta$-40 and -42 levels in the APP^Swe/+^ CM were much lower, around 1000 pg/mL for A $\beta$-40 and 100 pg/mL for A $\beta$-42. It is possible that natural production by the hiPSC-derived neurons confers increased A $\beta$ toxicity, or that metabolites other than A $\beta$ produced as a result of the Swedish mutation are responsible for the barrier disrupting effects on iBECs. Interestingly, the presence of another familial AD-causing mutation in presenilin-1 or -2 in iBECs is not only associated with reduced barrier function, but also with impaired glycolysis [9]. As a future direction, iBEC metabolism could be evaluated after CM treatment to elucidate potential metabolic mechanisms driving the specific vulnerability of iBECs on day 2 post-subculture, providing insight into the relation between BEC proliferation and BBB disruption in the context of AD. Our findings highlight that CM from iPSC-neurons can be used to study interactions of neuronal secretions with iBECs in context of AD. An additional future direction of this work could involve the co-culturing of APP^Swe/−^ iPSC neurons with iBECs [69] to more accurately model the close proximity of neurons with BECs in vivo.

Both the barrier and the interface functions of the BBB are influenced by BEC proliferation. Maturation of the brain vasculature into the stable adult BBB depends on the establishment and maintenance of a predominantly quiescent BEC monolayer. On the other hand, aberrant brain angiogenesis caused by inappropriate BEC proliferation is observed in pathologies such as Alzheimer’s disease (AD), glioblastoma multiforme, and cerebrovascular malformations [70–72]. A recent single-cell transcriptome analysis revealed that a subpopulation of angiogenic ECs is induced in the AD brain [73]. A central role for angiogenic vessels in the progression of AD is suggested by their presence in brain regions affected by AD pathology [70] and raises the possibility of inhibiting angiogenesis therapeutically [74]. A better understanding of the differential regulatory mechanisms of BBB functions in proliferating vs. quiescent BECs would inform novel therapeutic strategies to target vascular abnormalities and BEC activation in AD and other neurological diseases.

## Conclusion

iBECs derived from the GM25256 iPSC line offer a model to study BBB-specific expressional and functional changes that occur as BEC proliferation declines.

## Supplementary Information

**Additional file 1: Fig. S1.** Cell density of GM25256 iBECs at days 2 and 9 after subculture. Immunofluorescence analysis of DAPI + area in iBECs with no MC on days 2 and 9 after subculture. Each data point represents the average of 3–4 random fields of view per well. All data points obtained in the BrdU incorporation assays are represented. ***p < 0.001 (Unpaired two-tailed t-test). Means are displayed with their SE.

**Additional file 2: Fig. S2.** Effects of bFGF/RA treatment on BrdU incorporation in GM25256 iBECs. Immunofluorescence analysis of BrdU + area/DAPI + area in iBECs with no MC on days 2 and 9 post-subculture vs. on day 9 following treatment with 20 ng/mL bFGF and 10 $\mu$M RA on day 8. Each data point represents the average of 4 random fields of view per well. ***p < 0.001 (One-way ANOVA with Tukey’s multiple comparisons test). Means are displayed with their SE.

**Additional file 3: Fig. S3.** Western blot of GLUT1 in GM25256 iBECs on days 2 and 9 after subculture vs. in primary human astrocytes. Primary human astrocytes (Sciencell, cat no. 1800) were maintained on plates coated with poly-L-lysine in astrocyte medium (AM) (Sciencell, cat no. 1801), and proteins were extracted using protocol outlined in the Methods section.

**Additional file 4: Fig. S4.** Quality control experiments for glucose transport kinetic studies. **a-b.** Luminal-to-abluminal and abluminal-to-luminal permeability-surface area coefficients (Pe) for ^99m^Tc-DTPA on days 2 and 9 after subculture. ^99m^Tc-DTPA Pe was assayed simultaneously with ^14^C-DG Pe in the saturability experiments (Fig. 4a, b). ###p < 0.001 (One-way ANOVA with Tukey’s multiple comparisons test). **c.** The effects of 55.5 mM glucose vs. 55.5 mM mannitol on luminal-to-abluminal ^14^C-DG Pe. One differentiation was performed with n = 4 transwells per group. ***/###p < 0.001 (One-way ANOVA with Tukey’s multiple comparisons test). **d.** The effect of 2 μM BAY-876 (GLUT1 inhibitor) on luminal-to-abluminal ^14^C-DG Pe on days 2 and 9 after subculture. Two independent differentiations were performed with n = 4–5 transwells per group. ^14^C-DG Pe values were normalized to the uninhibited Pe on day 2. ***/###p < 0.001 (Two-way ANOVA with Tukey’s multiple comparisons test). **e.** Comparison of luminal-to-abluminal vs. abluminal-to-luminal permeability-surface area coefficients (Pe) for ^14^C-DG in the same differentiation of iBECs on day 2 after subculture. One differentiation was performed with n = 5 transwells per group. ***p < 0.001 (Two-way ANOVA with Tukey’s multiple comparisons test). **a-e.** Means are displayed with their SE.

## Data Availability

Data are available from the corresponding author with reasonable request.

## Abbreviations

hiPSC: Human induced pluripotent stem cell
BEC: Brain endothelial cell
iBECs: HiPSC-derived brain endothelial-like cells
BBB: Blood–brain barrier
TEER: Transendothelial electrical resistance
BrdU: 5-Bromo-2′-deoxyuridine (BrdU)
TJP and AJP: Tight and adherens junction proteins
GLUT1: Glucose transporter-1
TNF-α: Tumor necrosis factor alpha
CM: Conditioned medium
AD: Alzheimer’s disease
IFA: Immunofluorescence analyses
CLDN5: Claudin-5
VE-Cad: Vascular endothelial-cadherin
PECAM-1: Platelet endothelial cell adhesion molecule-1
ZO-1: Zona occludens-1 (ZO-1)
OCLN: Occludin
HESFM: Human endothelial serum-free medium
bFGF: Basic fibroblast growth factor
RA: Retinoic acid
PBS: Phosphate buffered saline
NAD: Normal antibody diluent
MFI: Mean fluorescence intensity
^14^C-DG: ^14^C-2-Deoxy-d-glucose
^99m^Tc-DTPA: ^99M^Tecnicium diethylenetriaminepentaacetic acid
APP: Amyloid precursor protein
Swe: Swedish mutation
ECs: Endothelial cells
NMC: No medium changes
SMC: Single medium change
CMC: Continual medium changes
PFKFB3: 6-Phosphofructo-2-kinase/fructose-2,6-biphosphatase 3
HK2: Hexokinase 2
MCT1: Monocarboxylate transporter-1
Pe: Permeability-surface area coefficient
A$$\beta$$: Amyloid beta
PTM: Post-translational modification
