# Supplementary figures and images for: Prolonged culturing of iPSC-derived brain endothelial-like cells is associated with quiescence, downregulation of glycolysis, and resistance to disruption by an Alzheimer’s brain milieu
Source: Fluids Barriers CNS. 2022 Feb 5;19:10. doi: 10.1186/s12987-022-00307-1 (PMC8817611; doi:10.1186/s12987-022-00307-1)

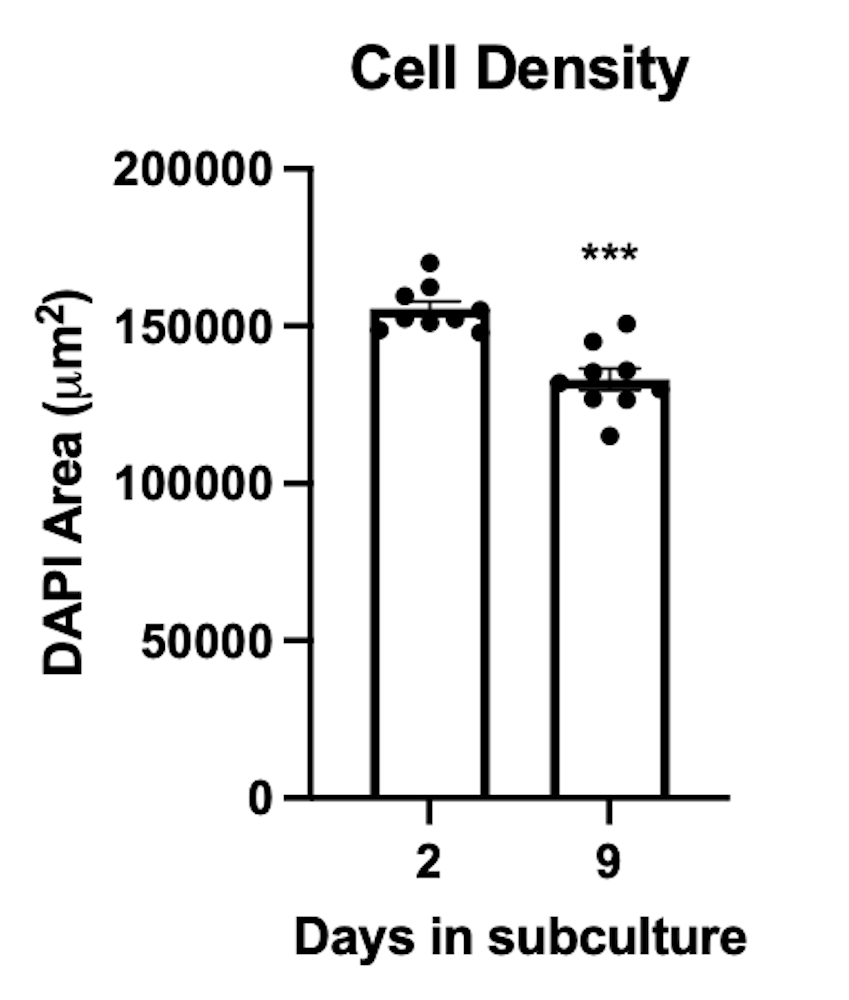

Supplement: Supplementary file 1 — Additional file 1: Fig. S1. Cell density of GM25256 iBECs at days 2 and 9 after subculture. Immunofluorescence analysis of DAPI + area in iBECs with no MC on days 2 and 9 after subculture. Each data point represents the average of 3–4 random fields of view per well. All data points obtained in the BrdU incorporation assays are represented. ***p < 0.001 (Unpaired two-tailed t-test). Means are displayed with their SE. [file 12987_2022_307_MOESM1_ESM.png]

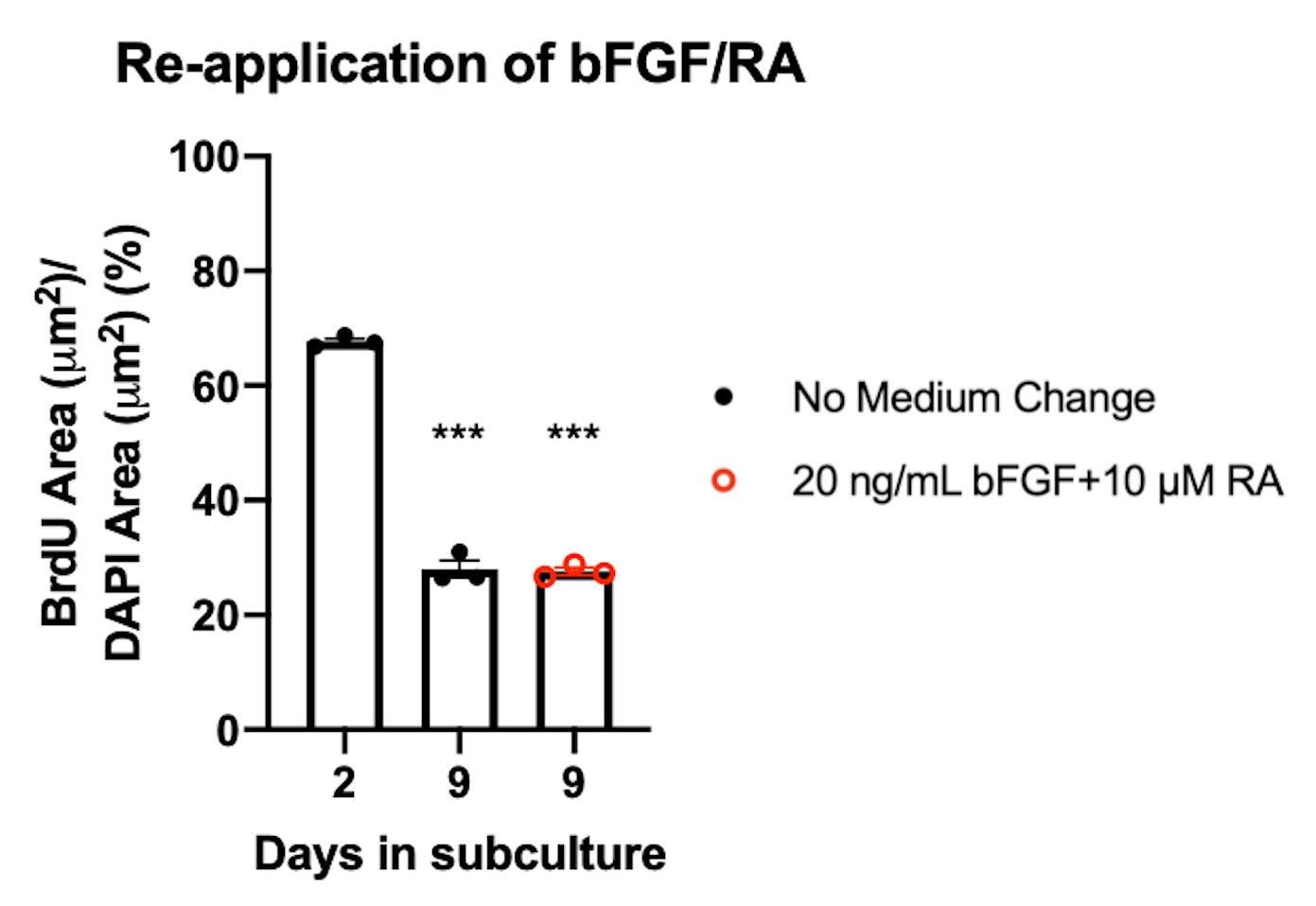

Supplement: Supplementary file 2 — Additional file 2: Fig. S2. Effects of bFGF/RA treatment on BrdU incorporation in GM25256 iBECs. Immunofluorescence analysis of BrdU + area/DAPI + area in iBECs with no MC on days 2 and 9 post-subculture vs. on day 9 following treatment with 20 ng/mL bFGF and 10 $\mu$μM RA on day 8. Each data point represents the average of 4 random fields of view per well. ***p < 0.001 (One-way ANOVA with Tukey’s multiple comparisons test). Means are displayed with their SE. [file 12987_2022_307_MOESM2_ESM.png]

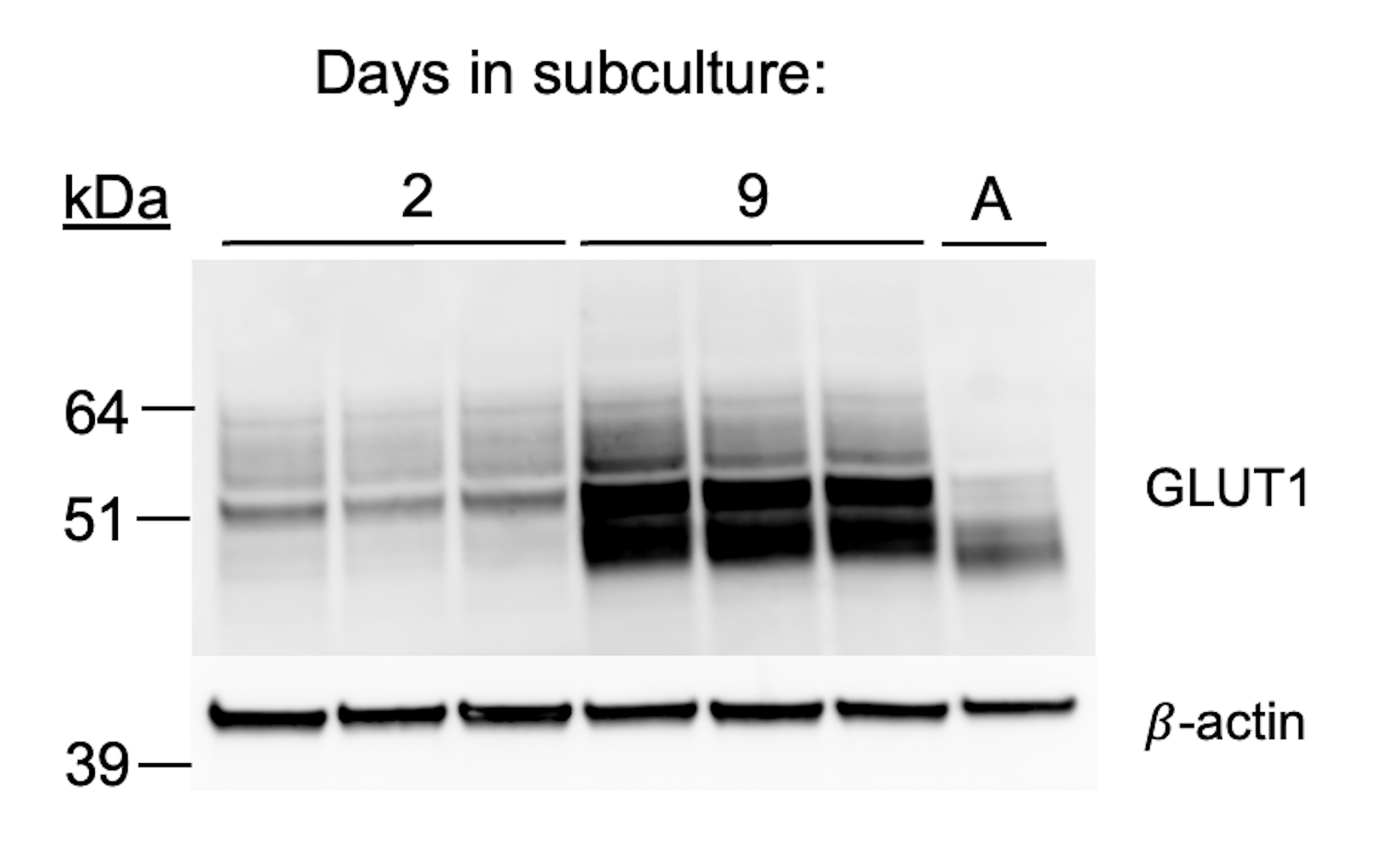

Supplement: Supplementary file 3 — Additional file 3: Fig. S3. Western blot of GLUT1 in GM25256 iBECs on days 2 and 9 after subculture vs. in primary human astrocytes. Primary human astrocytes (Sciencell, cat no. 1800) were maintained on plates coated with poly-L-lysine in astrocyte medium (AM) (Sciencell, cat no. 1801), and proteins were extracted using protocol outlined in the Methods section. [file 12987_2022_307_MOESM3_ESM.png]

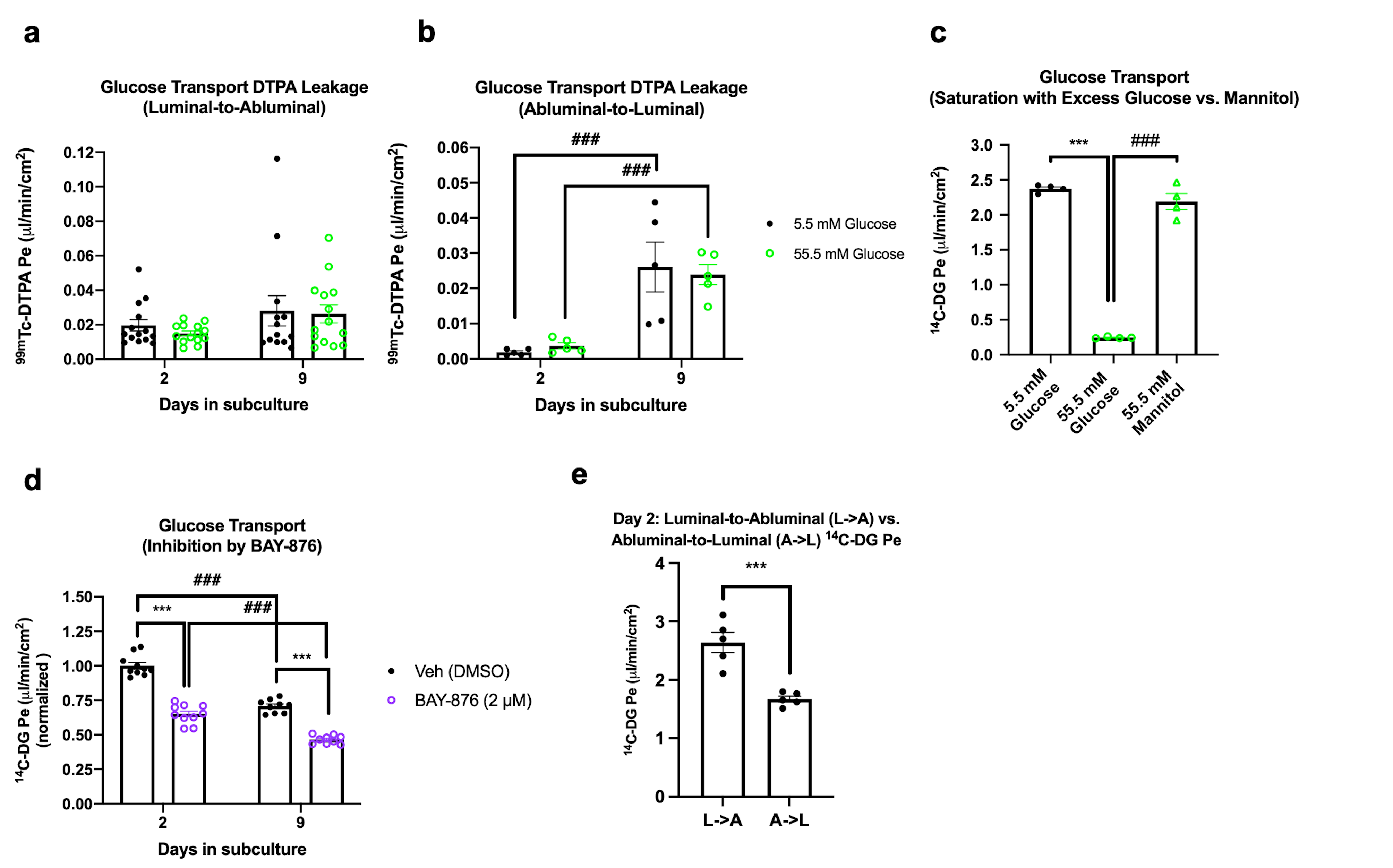

Supplement: Supplementary file 4 — Additional file 4: Fig. S4. Quality control experiments for glucose transport kinetic studies. a-b. Luminal-to-abluminal and abluminal-to-luminal permeability-surface area coefficients (Pe) for 99mTc-DTPA on days 2 and 9 after subculture. 99mTc-DTPA Pe was assayed simultaneously with 14C-DG Pe in the saturability experiments (Fig. 4a, b). ###p < 0.001 (One-way ANOVA with Tukey’s multiple comparisons test). c. The effects of 55.5 mM glucose vs. 55.5 mM mannitol on luminal-to-abluminal 14C-DG Pe. One differentiation was performed with n = 4 transwells per group. ***/###p < 0.001 (One-way ANOVA with Tukey’s multiple comparisons test). d. The effect of 2 μM BAY-876 (GLUT1 inhibitor) on luminal-to-abluminal 14C-DG Pe on days 2 and 9 after subculture. Two independent differentiations were performed with n = 4–5 transwells per group. 14C-DG Pe values were normalized to the uninhibited Pe on day 2. ***/###p < 0.001 (Two-way ANOVA with Tukey’s multiple comparisons test). e. Comparison of luminal-to-abluminal vs. abluminal-to-luminal permeability-surface area coefficients (Pe) for 14C-DG in the same differentiation of iBECs on day 2 after subculture. One differentiation was performed with n = 5 transwells per group. ***p < 0.001 (Two-way ANOVA with Tukey’s multiple comparisons test). a-e. Means are displayed with their SE. [file 12987_2022_307_MOESM4_ESM.png]
